# Emergency presentations following youth suicide attempts during the COVID-19 pandemic: temporal trends and associations with containment measures

**DOI:** 10.1007/s00787-026-03119-y

**Published:** 2026-07-04

**Authors:** Johannes Schmier, Paul L. Plener, Diana Klinger

**Affiliations:** 1 Department of Child and Adolescent Psychiatry, Medical University of Vienna, Vienna, Austria; 2 Comprehensive Center for Pediatrics, Medical University of Vienna, Vienna, Austria; 3 Department of Child and Adolescent Psychiatry and Psychotherapy, University of Ulm, Ulm, Germany

**Keywords:** COVID-19 pandemic, Suicide attempt, Psychiatric emergency presentations, Adolescents

## Abstract

The COVID-19 pandemic has raised concerns about adverse effects on youth mental health, including suicidal behavior. While suicide mortality remained stable in many countries, less is known about temporal changes in suicide attempts presenting to clinical services. This study examined trends in psychiatric emergency presentations following suicide attempts among children and adolescents across COVID-19 pandemic phases and their association with the intensity of governmental containment measures. A retrospective analysis was conducted at a tertiary care center in Vienna, Austria, between January 2019 and December 2023, distinguishing pre-pandemic, pandemic, and post-pandemic periods. COVID-19 containment stringency was quantified using the Oxford COVID-19 Government Response Tracker Stringency Index. Temporal changes in monthly suicide attempt presentations were analyzed using Poisson regression models, adjusting for linear time trends. A total of 6,319 psychiatric emergency presentations were recorded, including 722 presentations following suicide attempts (11.4%). Compared with the pre-pandemic period, suicide attempt-related presentations increased significantly during the pandemic (RR = 1.80, *p* < .001), while the post-pandemic period did not differ significantly from pre-pandemic levels. Higher monthly suicide attempt presentation rates were associated with greater containment stringency (RR per point = 1.007, *p* < .001). An independent upward temporal trend in suicide attempt presentations was observed across the study period. Associations with policy stringency and persistent temporal trends suggest that suicide attempts may serve as an indicator of psychosocial burden not captured by mortality data alone. Continuous monitoring of emergency presentations and access to low-threshold mental health support are necessary beyond periods of acute crisis.

## Introduction

Suicide among adolescents is a preventable cause of premature death and represents a major public health concern [1]. It has been shown that previous suicide attempts increase the risk of later completed suicide and significantly reduce life expectancy. Men who attempt suicide for the first time at the age of 20 lose, on average, around 18 years, while those attempting at age 50 lose approximately 10 years. For women, the reduction is around 11 and 8 years, respectively [2]. A meta-analysis comprising data from different age groups showed that 1, 5 and 10 years after a non-fatal suicide attempt, the suicide rates of those affected were 2.8%, 5.6% and 7.4% [3]. While these findings should be applied to minors with caution, adolescent-specific evidence also demonstrates clinically relevant long-term risk: in a population-based cohort study of adolescents discharged from hospital after a first self-poisoning episode, suicide risk was markedly increased compared with matched reference individuals, identifying hospital-treated self-poisoning in adolescence as an important marker for later suicide and premature death [4]. Given their strong predictive value for completed suicide [5], monitoring suicide attempts provides an important opportunity for early intervention and sustained secondary prevention.

The suicide rate in Austria in 15–19-year-old ones is above the European average, when looking in the latest available data from 2022 [6]. Globally, the suicide rate in 10–19-year-old ones in Austria is below the average of 3.77/100,000 in the WHO mortality database [7]. Despite individual years with rising incidences, the number of deaths by suicide in the general population has been declining in recent decades. National suicide mortality data are reported regularly, yet systematic data on suicide attempts remain lacking [8], although suicide attempts are a key indicator of later suicide risk [2, 5]. At the same time, this indicator should be interpreted within the broader context of adolescent suicide risk, as most adolescents who attempt suicide will not die by suicide, and among adolescents who die by suicide, a prior non-fatal attempt is not always documented [9].

Adolescence represents a particularly vulnerable developmental phase for the onset of suicidal thoughts and behaviors. It has been shown that adolescents who reported any form of suicidal thoughts or behavior before the age of 16 show higher rates of anxiety disorders, suicidal thoughts and suicidal behavior, as well as generally poorer levels of functioning in adulthood. These early vulnerabilities are further reflected in the finding that children and adolescents with suicidal thoughts and behaviors are at risk of having a disrupted transition to adulthood [10].

Gender differences in suicidal behavior are well documented: the number of suicide attempts among female adolescents is significantly higher than among their male peers, but the ratio is reversed when it comes to suicides [11]. Similar gender differences have been observed among young people presenting with acute mental health crises, with an overrepresentation of female adolescents among emergency hospital presentations [12, 13].

During the COVID-19 pandemic, reductions in well-being and increases in mental health problems were reported both in Austria and internationally [14–17]. In Austria, the pandemic was associated with impaired mental health among Austrian schoolchildren. Mental well-being and life satisfaction were significantly lower in 2021 than in 2018. Depressive symptoms, anxiety symptoms, sleep problems and eating disorders were higher than before the beginning of the COVID-19 pandemic. Approximately one-third of participants reported suicidal thoughts [15]. These findings were confirmed and extended by subsequent surveys in Austria. A study conducted at the end of 2021 found that 62% of girls and 38% of boys exceeded the thresholds for clinically relevant depressive symptoms, 49% of girls and 29% of boys exceeded those of symptoms for anxiety, and 28% of girls and 17% of boys exceeded those for sleep disorders. The prevalence of suicidal thoughts during the two weeks prior to the survey was 47% among girls and 32% among boys [16]. Longitudinal population-based data from Germany further demonstrated that the prevalence of mental health problems and anxiety among children and adolescents increased during the early pandemic phase and remained elevated across multiple waves until late 2021, with only partial recovery. Adolescents from socioeconomically disadvantaged families and those with parental mental health problems were identified as particularly vulnerable [17].

At the beginning of the COVID-19 pandemic, concerns were raised about a possible increase in suicide rates [18, 19], including among youth [20]. These concerns shaped early public health discussions about the potential mental health consequences of the pandemic. However, suicide mortality, suicidal ideation, self-harm, and non-fatal suicide attempts represent related but distinct outcomes that may follow different temporal patterns. For example, an analysis of police data from three German federal states found no increase in suicide rates among children, adolescents, and young adults during the pandemic period [21]. Overall, international findings on suicidality and self-harm among children and adolescents have been heterogeneous. Several studies reported declines in overall psychiatric emergency presentations among children and adolescents and reductions in self-harm or suicidality, especially during the initial lockdown periods [22–25]. Consistent with these findings, a German multi-site study comparing adolescents assessed before and shortly after the first school lockdown found no increase in emotional or behavioral problems, suicidal thoughts, or suicide attempts and even reported a reduction in suicide plans during the early lockdown phase [26]. Meta-analytic evidence also indicated overall reductions in pediatric emergency department visits for most mental-health-related presentations during the early pandemic phase [27]. Other studies, including also later phases of the pandemic described increases, either in the absolute number of presentations related to suicidality or in the proportion of such presentations among all psychiatric emergencies. For instance, increases in suicidality or suicide attempts during parts of the pandemic were reported in Italy [28], in US pediatric emergency departments [20, 29], in Spain [30, 31], in Slovenia [32], and in Germany [33], with a meta-analysis of 42 studies from pediatric emergency departments underlining this increase [27]. Furthermore, a time-series study from France reported a distinct temporal pattern, namely a decrease in suicide attempts among children during the first lockdown, followed by a substantial increase later in 2020, independent of seasonal or long-term trends [34]. In Austria, suicide mortality showed a marked increase in 2022, with stabilization and a small reduction since then [8]; however, multiple centers reported rising referrals and admissions following suicide attempts. This was observed in pediatric intensive care units and emergency departments, as well as in child and adolescent psychiatric clinics [35]. Since 2021 and up to the end of 2023, inquiries regarding intentional self-poisoning in Austria have increased year on year. The number of patients aged 10–19 with the additional diagnosis of suicide attempt or deliberate self-harm during inpatient stays was higher in 2021 and 2022 than in 2019 and 2020, although a decrease was observed again in the first half of 2023 [36]. Together, these heterogeneous findings make it difficult to determine whether pandemic-related shifts in youth suicidal behavior were temporary or reflected more persistent changes.

In addition, previous research has indicated that the intensity of governmental containment measures may have influenced the mental health burden [37, 38]. Periods with stricter restrictions, including school closures and reduced social contact, have been associated with higher psychological distress and increased rates of self-harm and suicidality in youth [39, 40]. This raises the question of whether the strictness of public health measures is related to the frequency of suicide attempts presenting to clinical services. The strictness of governmental containment policies implemented to limit viral transmission can be quantified using the COVID-19 Stringency Index, based on data from the Oxford COVID-19 Government Response Tracker (OxCGRT; [41]). Taken together, these findings underscore the need to investigate temporal trends in suicide attempts across both the pandemic and post-pandemic periods, and to examine whether changes in suicide attempts are associated with the severity of COVID-19–related public health measures. To address these gaps, we formulated the following hypotheses:

1. We hypothesized an increase in clinical presentations related to suicide attempts starting at the beginning of the pandemic.
2. We hypothesized a decrease in clinical presentations related to suicide attempts starting at the end of the pandemic.
3. We hypothesized a positive association between clinical presentations related to suicide attempts and COVID-19 Stringency Index.

## Methods

### Sample and setting

A retrospective analysis of all psychiatric emergency presentations between 2019 and 2023 was conducted at the Department of Child and Adolescent Psychiatry of the Medical University of Vienna, Austria. The department serves as the designated emergency child and adolescent psychiatric service for eleven of twenty-three districts of Vienna, covering a catchment area of approximately 835,000 inhabitants (as of January 1, 2024) [42]. It provides 24/7 emergency psychiatric care and is one of the two child and adolescent psychiatric services in Vienna with continuous round-the-clock emergency responsibility. However, adolescents may present in emergency situations or be transferred from pediatric services regardless of catchment area.

Children and adolescents up to the age of 18 years who presented to the emergency outpatient service were included in the study. Based on the list of all outpatient visits without appointments compiled by the ambulance staff and the medical documentation of these visits in the clinic’s patient management program, data including age, gender and information regarding suicide attempts (yes/no) were collected from the documentation written by medical staff. Patients were classified according to legal gender as recorded in the patient management system. This administrative variable usually corresponds to sex assigned at birth but reflects the affirmed legal gender in cases in which legal gender recognition had occurred. Multiple visits on the same day were combined, and visits that were not classified as emergency visits were excluded. This applies to presentations in which there was no medical contact with the patient (e.g., the patient left before assessment) or to follow-up visits arranged at short notice (e.g., an acute visit the day before). The study received approval from the Ethics Committee of the Medical University of Vienna (no. 1744/2021).

### Measures

#### Suicide attempt

A suicide attempt was defined as any action or sequence of actions initiated with the intention of ending one’s own life but not resulting in death [43]. Classification was based on the clinical emergency documentation, including patients’ self-report of suicidal intent. Presentations involving self-harm without documented suicidal intent were not classified as suicide attempts. Patients were asked about the last suicide attempt before the current emergency consultation, and presentations were classified as suicide attempt-related if this reported attempt had not already been previously recorded before the current consultation. No assessment of the danger or severity of the attempt was undertaken.

#### Pandemic period

To examine temporal effects related to the COVID-19 pandemic, a three-level categorical pandemic variable was created. The pre-pandemic period comprised all months up to February 2020. The pandemic period covered March 2020 through June 2023, during which varying restrictions were in place. The post-pandemic period was defined as beginning in July 2023, coinciding with the full discontinuation of COVID-19-related measures in Austria [44].

#### Stringency index of the OxCGRT

In addition, the study included the OxCGRT Stringency Index to quantify the intensity of governmental containment measures [41]. The Stringency Index is a composite measure ranging from 0 to 100 that reflects policy strictness across domains such as school closures, travel restrictions, and public gathering regulations. For each month, the mean of all daily Stringency Index values from Austria was calculated. Values were set to zero for all months prior to February 2020. Monthly means from February 2020 through December 2022 were used as provided. Between January 1 and June 30, 2023, the last available value (35.19), which had remained unchanged since June 2022, was carried forward. Beginning July 1, 2023, the index was set to zero again, as all COVID-19 measures had ceased [44].

### Data analysis

Statistical analyses were conducted using IBM SPSS Statistics (Version 30). Age, gender, monthly counts of psychiatric emergency presentations and presentations following suicide attempts were summarized for the study period from January 2019 to December 2023. Categorical variables were reported as frequencies and percentages, and continuous variables as means and standard deviations.

To examine temporal changes in suicide attempt-related presentation rates, Poisson regression models with a log link function were performed. The monthly number of all psychiatric emergency presentations was included as an offset. In the first model, the pandemic period (pre-pandemic, pandemic, post-pandemic) was included as a categorical predictor, with the pre-pandemic period serving as the reference category. Time in months was included as a continuous predictor to account for linear temporal trends across the five-year observation period. In the second model, the monthly OxCGRT Stringency Index was entered as a continuous predictor. Time in months was again entered as a covariate to estimate changes independent of pandemic-related restrictions. Model fit was assessed using goodness-of-fit statistics. Potential overdispersion was evaluated by inspecting deviance/df and Pearson *χ*²/df values, with values close to 1 indicating no relevant overdispersion. Zero inflation was assessed descriptively by examining the monthly suicide attempt counts. Statistical significance was set at *p* < .05.

## Results

Table 1 presents the descriptive characteristics of the study sample, including age, gender distribution, monthly counts of psychiatric emergency presentations, and monthly counts of presentations following suicide attempts for the period from January 2019 to December 2023.

**Table 1:** Descriptive statistics for psychiatric emergency presentations and presentations following suicide attempts by year (2019–2023)

Variable	2019	2020	2021	2022	2023	Total
**Total emergency presentations, n**	1279	1028	1309	1260	1443	6319
Age (years), *M* (*SD*)	14.6 (2.4)	14.8 (2.2)	14.6 (2.4)	14.3 (2.3)	14.4 (2.2)	14.5 (2.3)
Gender						
Female, n (%)	870 (68.0%)	710 (69.1%)	937 (71.6%)	992 (78.7%)	1163 (80.6%)	4672 (73.9%)
Male, n (%)	409 (32.0%)	318 (30.9%)	372 (28.4%)	268 (21.3%)	280 (19.4%)	1647 (26.1%)
**Suicide attempts, n (%)**	67 (5.2%)	103 (10.0%)	168 (12.8%)	200 (15.9%)	184 (12.8%)	722 (11.4%)
Gender						
Female, n (%)	64 (95.5%)	87 (84.5%)	152 (90.5%)	180 (90%)	167 (90.8%)	650 (90%)
Male, n (%)	3 (4.5%)	16 (15.5%)	16 (9.5%)	20 (10%)	17 (9.2%)	72 (10%)

**Fig. 1:**
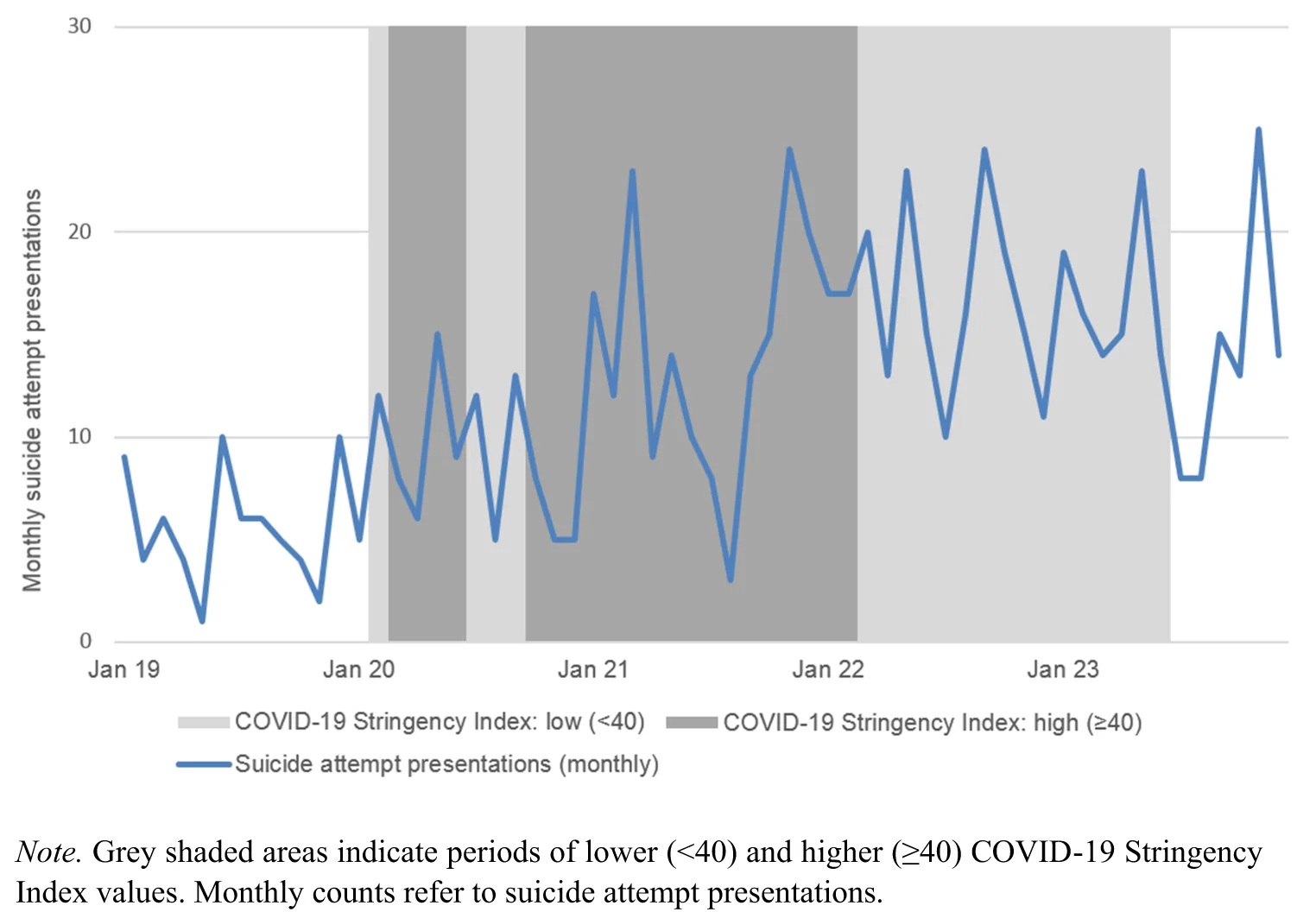
Monthly suicide attempt presentations and COVID-19 stringency levels, 2019–2023

Figure 1 illustrates monthly suicide attempt presentations between January 2019 and December 2023 in relation to periods of lower and higher COVID-19 stringency. The figure shows pronounced temporal variation, with higher numbers of suicide attempt-related presentations during phases of increased containment stringency and a sustained elevation over time.

Table 2 summarizes the results of the two Poisson regression models examining predictors of monthly suicide attempt-related presentations relative to all psychiatric emergency presentations. Model diagnostics indicated no evidence of relevant overdispersion. Monthly suicide attempt counts ranged from 1 to 25, with no zero-count months, indicating no evidence of zero inflation.

### Model 1: Pandemic period

The Poisson regression model including the three pandemic periods showed a significant overall effect (likelihood-ratio *χ*²(3) = 76.64, *p* < .001). Compared with the pre-pandemic period, the rate ratio for suicide attempt presentations relative to all psychiatric emergency presentations was significantly higher during the pandemic phase. In the post-pandemic period, the suicide attempt-related presentation rate ratio was 1.25 times higher than before the pandemic; however, this difference was not statistically significant. Pairwise comparisons indicated that the rate of suicide attempts-related presentations was significantly higher during the pandemic than during the pre-pandemic period and also higher than during the post-pandemic period (*p* = .004). No significant difference was observed between the pre- and post-pandemic periods. Time (in months) was positively associated with suicide attempt-related presentation rates, corresponding to a modelled average monthly increase of approximately 1% within the observation period (see Table 2).

### Model 2: COVID-19 Stringency Index

The Poisson regression model including the monthly COVID-19 Stringency Index also showed a significant overall effect (likelihood-ratio *χ*²(2) = 70.08, *p* < .001). The Stringency Index was a significant predictor of monthly suicide attempt-related presentation rates, indicating an increase of approximately 0.7% in suicide attempt presentations per additional point on the index. Time was again positively associated with suicide attempt-related presentation rates, corresponding to a modelled average monthly increase of approximately 1.6% within the observation period.

**Table 2:** Poisson regression models predicting monthly emergency suicide attempt presentations

Predictor	RR	95% CI		*p*
		LL	UL	
**Model 1: Pandemic period**				
Constant	0.052	0.042	0.065	.000
Pre-pandemic period (ref)	1			
Pandemic period	1.796	1.310	2.462	< .001
Post-pandemic period	1.251	0.776	2.016	.359
Time (months)	1.010	1.003	1.018	.007
**Model 2: COVID-19 stringency index**				
Constant	0.052	0.042	0.065	.000
Stringency Index (per point)	1.007	1.004	1.010	< .001
Time (months)	1.016	1.011	1.021	< .001

*Note. CI* = confidence interval, *LL* = lower limit, *UL* = upper limit

Goodness-of-fit diagnostics: Model 1: deviance/df = 1.23, Pearson *χ*²/df = 1.18; Model 2: deviance/df = 1.33, Pearson *χ*²/df = 1.27

## Discussion

In this retrospective study of child and adolescent psychiatric emergency presentations in Austria between 2019 and 2023, we observed a marked increase in presentations following suicide attempts during the COVID-19 pandemic. Although overall emergency admissions temporarily declined in 2020, suicide attempt-related presentations increased during the pandemic phase and remained elevated thereafter, without a return to pre-pandemic levels in the post-pandemic period. These changes were associated both with the pandemic period itself and with the intensity of governmental containment measures, with higher suicide attempt presentation rates observed during periods of greater restriction. In addition to these pandemic-related effects, a positive time coefficient was observed. This indicates an upward trend in suicide attempt-related emergency presentations across the 2019–2023 observation period, but should not be interpreted as evidence of a stable long-term trajectory beyond the study period.

These findings are consistent with international evidence indicating heterogeneous, phase-specific changes in youth suicidality during the COVID-19 pandemic. Several studies reported reductions in psychiatric emergency presentations and suicidality during the early lockdown phases, which have been interpreted as reduced help-seeking, limited access to services, and avoidance of emergency departments rather than true reductions in distress [22, 23, 26]. Meta-analytic evidence similarly demonstrated an overall decrease in pediatric emergency department visits for most mental health presentations during the initial pandemic phase [27].

In contrast, studies covering later phases of the pandemic reported increases in suicide attempts or suicide-related emergency presentations, particularly among adolescents, pointing to delayed or cumulative effects of prolonged psychosocial stressors. Such patterns have been observed in France, where an initial decline in suicide attempts during the first lockdown was followed by an increase later in 2020 [34], as well as in Germany, the United States, and other countries reporting rising rates of suicide attempts among youth as the pandemic progressed [20, 29, 33]. Our results are consistent with this latter body of evidence and extend it by demonstrating that elevated suicide attempt presentations persisted beyond the acute pandemic phase. In this respect, the present findings support the first hypothesis of an increase in suicide attempt presentations during the pandemic, while providing only limited support for a post-pandemic decline. Rather than indicating a clear return to pre-pandemic levels, the persistence of elevated rates into 2023 suggests that changes in suicide attempt-related emergency presentations may extend beyond the acute pandemic phase. By combining a multi-year observation period with explicit modeling of pandemic phases and containment stringency, our study helps to contextualize previous findings by highlighting the importance of timing and policy context when interpreting pandemic-related changes in youth suicidal behavior.

The observed association between suicide attempt presentations and the COVID-19 Stringency Index suggests that the intensity of containment measures may be relevant for understanding temporal fluctuations in youth suicidality, without implying a causal relationship. The Stringency Index captures the overall strictness of governmental responses, including school closures, restrictions on social contact, and limitations to public life, and can therefore be interpreted as a proxy for broader psychosocial stressors experienced by children and adolescents during the pandemic, all of which have been linked to increased psychological distress [39, 40]. School-related factors may also have shaped emergency presentation patterns, as youth mental-health-related emergency department visits have been shown to vary across the school calendar [45]. School holidays, closures, and remote schooling altered daily routines, peer contact, and access to school-based support and referral pathways. In our study, school closures were partly reflected in the COVID-19 Stringency Index, while the five-year observation period allowed seasonal fluctuations to be considered across multiple school years. Taken together, higher policy stringency may therefore reflect both cumulative stress exposure and disruption of usual school-related structures, with help-seeking behavior increasingly shifting toward emergency psychiatric services once restrictions ease. Beyond these pandemic-related effects, we observed an upward temporal trend in suicide attempt presentations that was independent of both pandemic phase and governmental stringency. In this sense, the pandemic may have accelerated developments that were already underway, suggesting possible future trajectories if the ongoing stress experienced by young people is not adequately addressed. This interpretation is consistent with evidence of deteriorating youth mental health before the pandemic and with recent discussions of a broader youth mental health crisis and societal “megatrends” affecting young people’s mental health [46–48].

Our findings underline the relevance of child and adolescent psychiatric emergency services as a key indicator of youth mental health burden during and after societal crises. In particular, suicide attempts may respond more sensitively to psychosocial stressors than suicide mortality, which can remain stable despite substantial changes in acute distress and help seeking. From a clinical and public health perspective, routinely collected emergency presentation data may therefore function as an early warning system to detect shifts in clinically presenting severe youth mental health outcomes and to guide service planning and prevention. The persistent elevation of suicide attempt presentations beyond the acute pandemic phase suggests that monitoring should continue even after restrictive measures are lifted, and that low threshold, timely access to outpatient and school-based support is essential to prevent crisis escalation into emergency care. This is particularly relevant for vulnerable groups, including girls, and individuals with pre-existing mental health problems, who have been shown to be disproportionately affected by the psychological consequences of the pandemic and related containment measures. Evidence from adult psychiatric emergency settings from Germany similarly suggests that pandemic-related increases in suicide attempt presentations were particularly pronounced among clinically vulnerable groups, including patients with borderline personality disorder [49, 50]. In younger populations, such vulnerability may be further compounded by developmental and school-related factors, including peer relationships, family routines, and access to age-specific support structures.

The increase in the proportion of girls presenting to emergency services across the observation period highlights the importance of considering gender related patterns in help seeking and clinical presentation. This female predominance was even more pronounced among suicide attempt-related presentations: across the observation period, 90.0% of suicide attempt-related presentations were recorded among female patients. Although adolescents of both genders in Austria are generally affected by psychiatric disorders to a similar extent [51], this pronounced female predominance should be considered within the broader pattern of mental health burden among girls during the pandemic. In Austria, girls showed higher rates of depressive, anxiety, and sleep problems during this period [15], consistent with international evidence of stronger pandemic-related mental health impacts among girls [14, 52]. This likely contributes to, but does not fully explain, the observed sex differences in both overall and suicide attempt-related emergency presentations, pointing also to potential differences in pathways to care and service use. Finally, the association with policy stringency supports the need for health policy decisions to explicitly consider possible unintended mental health effects and to ensure that protective and accessible support structures for young people remain available during periods of disruption.

### Strengths and limitations

A strength of this study is the inclusion of all child and adolescent psychiatric emergency presentations within a large catchment area, ensuring complete coverage of clinically relevant acute contacts. The five-year observation period spanning pre-pandemic, pandemic, and post-pandemic phases allowed us to examine temporal patterns across these phases. In addition, the combination of routine clinical data with a standardized policy indicator enabled the examination of associations between suicide attempt presentations, pandemic phases, and containment stringency.

Several limitations should be considered. The monocentric design may limit generalizability to other healthcare settings or regions. The analysis was based on service-level presentation rates rather than population-based incidence rates, as youth can present in emergency situations or be transferred from pediatric services regardless of catchment area. We focused on suicide attempt-related presentations to ensure a clearly defined suicidality outcome. Non-suicidal self-injury (NSSI) was often present in these adolescents, but was not systematically extracted as a separate variable. Therefore, the findings refer specifically to emergency presentations involving documented suicidal intent and should not be generalized to NSSI or other forms of adolescent self-harm. Classification relied on clinical documentation and patients’ report of the last suicide attempt before the emergency consultation. Therefore, recall bias cannot be excluded, and the data do not capture lifetime history of suicide attempts. No information was available on the severity of suicide attempts or individual-level socioeconomic characteristics. The COVID-19 Stringency Index reflects containment measures at the national level and may not capture local variation in restrictions or individual exposure. Finally, due to the observational design, causal inferences regarding the effects of containment measures on suicide attempt presentations cannot be drawn.

## Conclusion

The present findings indicate that suicide attempt-related emergency presentations remained elevated beyond the acute phase of the COVID-19 pandemic and the lifting of restrictive measures. Both the pandemic period and the intensity of containment policies were associated with changes in suicide attempt presentations, underscoring the relevance of contextual and policy-related factors for youth mental health. Suicide attempt-related emergency presentations may provide clinically relevant information on psychological burden among young people that is not captured by mortality data alone.

Future research should build on these findings by incorporating multicenter designs and individual-level data to allow differentiation by age, gender, and vulnerability profiles. Continuous monitoring of suicide attempt presentations, extending beyond periods of acute crisis, is needed to inform early intervention, service planning, and policy decisions aimed at mitigating long-term mental health consequences among children and adolescents.

## Data Availability

The datasets used and/or analyzed during the current study are available from the corresponding author on reasonable request.
